# KPNB1-mediated nuclear import is required for motility and inflammatory transcription factor activity in cervical cancer cells

**DOI:** 10.18632/oncotarget.15834

**Published:** 2017-03-02

**Authors:** Tamara Stelma, Virna D. Leaner

**Affiliations:** ^1^ Division of Medical Biochemistry and Structural Biology, SAMRC Gynaecology Cancer Research Centre, Department of Integrative Biomedical Sciences, Faculty of Health Sciences, Institute of Infectious Diseases and Molecular Medicine, University of Cape Town, Cape Town, South Africa

**Keywords:** karyopherin/importin, cervical cancer, chemotherapeutic, nuclear import, inflammation

## Abstract

Karyopherin β1 is a nuclear import protein involved in the transport of proteins containing a nuclear localisation sequence. Elevated Karyopherin β1 expression has been reported in cancer and transformed cells and is essential for cancer cell proliferation and survival. Transcription factors such as NFĸB and AP-1 contain a nuclear localisation sequence and initiate the expression of multiple factors associated with inflammation and cancer cell biology. Our study investigated the effect of inhibiting nuclear import via Karyopherin β1 on cancer cell motility and inflammatory signaling using siRNA and the novel small molecule, Inhibitor of Nuclear Import-43, INI-43. Inhibition of Karyopherin β1 led to reduced migration and invasion of cervical cancer cells. Karyopherin β1 is essential for the translocation of NFĸB into the nucleus as nuclear import inhibition caused its cytoplasmic retention and decreased transcriptional activity. A similar decrease was seen in AP-1 transcriptional activity upon Karyopherin β1 inhibition. Consequently reduced interleukin-6, interleukin-1 beta, tumour necrosis factor alpha and granulocyte macrophage colony stimulating factor expression, target genes of NFkB and AP-1, was observed. Migration studies inhibiting individual transcription factors suggested that INI-43 may affect a combination of signaling events. Our study provides further evidence that inhibiting KPNB1 has anti-cancer effects and shows promise as a chemotherapeutic target.

## INTRODUCTION

The Karyopherin beta superfamily forms the major class of soluble transport proteins that transport proteins larger than 20-40kDa through the nuclear pore complex (NPC). Karyopherin β1 (KPNB1) is the predominant nuclear import protein and transports cargo into the nucleus independently (non-classical pathway) or through the association with a karyopherin adaptor protein (classical pathway) [1]. In the classical import pathway cargo is identified by a Karyopherin alpha protein through a nuclear localisation sequence (NLS) and binds forming a dimer. Together this dimer then binds KPNB1 and is allowed access through the NPC as a trimeric complex [2–4]. In the non-classical pathway KPNB1 is able to directly recognise a specific NLS on the cargo, binding to it. The nuclear transport system plays a critical role in normal cell functioning and therefore it isn't unusual that its dysregulation has been associated with carcinogenesis. KPNB1 expression has been found to be upregulated in various cancers including cervical cancer and correlates with a poor patient prognosis in gastric cancer [5, 6].

One of the classical hallmarks of cancer is the ability of cancer cells to invade tissue surrounding the site of the primary tumour and metastasize to a secondary location [7]. This occurrence is responsible for the majority of cancer-related deaths. When Hanahan and Weinberg (2011) revisited the classical hallmarks they noted that an inflammatory microenvironment plays an important role in sustaining many of the other hallmarks of cancer including the ability of cancer cells to invade [8]. The presence of chemokines and cytokines in the tumour microenvironment, whether produced by cancer cells or infiltrating immune cells, directly contribute to the activation of transcription factors such as NFkB and AP-1 [9–11]. TNF-α is a cytokine commonly known to activate both NFkB and AP-1 [12]. The activation of such transcription factors initiates the expression of various target genes contributing to cancer progression by enhancing proliferation (IL-1, TNF-α, GM-CSF, Cyclin D1), evading apoptosis (BCL2, TRAF2, BCL3) and promoting cell invasion (IL-1, IL-6, TNF, MMPs) [13–20].

The NFĸB family consists of 5 members of which p65 and p50 are activated in the canonical activation pathway in an inflammatory response. Activation and translocation of the transcription factor into the nucleus can be induced in most cell types but some cells such as macrophages and tumour cells can have constitutively active NFĸB. Activation can be stimulated by several events including the presence of cytokines or artificially in response to the phorbol ester, phorbol-12-myristate-13-acetate (PMA) [21, 22]. It has been identified that NFκB's p65 and p50 subunits have a classical NLS, suggesting that they require KPNB1 for their nuclear import [23]. No mutations in the NFĸB gene or its inhibitors have been associated with cancer but rather the increased presence of activated NFĸB has been associated with an increase in inflammation promoting carcinogenesis [10, 24–27].

Activator protein 1 (AP-1) is a transcription factor consisting of dimers of mainly JUN and FOS family members. Much like NFkB it is activated by inflammatory cytokines and growth factors as well as the phorbol ester, PMA. AP-1 regulates genes involved in many cellular processes including; proliferation, differentiation, apoptosis, angiogenesis and tumour invasion. Increased expression of JUN and FOS family members is associated with several cancers [28–30]. Nuclear import of AP-1 is suggested to be mediated through the non-classical nuclear import pathway as KPNB1 was found to have a significantly greater affinity for AP-1 than Karyopherin alpha and therefore is reported to be transported into the nucleus by KPNB1 alone [31].

As NFkB and AP-1 play such a pivotal role in inflammatory signaling and promoting the cancer phenotype, inhibiting their function could have chemotherapeutic benefits [32–36]. The small molecule inhibitor of KPNB1, INI-43, has been described by van der Watt *et al*. (2016) to interfere with nuclear import of transcription factors such as NFAT. Nuclear import inhibition through KPNB1 has also previously been shown in our laboratory and by others to have anti-cancer potential by inhibiting cancer cell proliferation and inducing cell death by apoptosis *in vitro* as well as inhibiting tumour growth *in vivo* [5, 37–39]. As both NFĸB and AP-1 require KPNB1 for their nuclear translocation we hypothesized that inhibiting KPNB1 could inhibit the activity of these transcription factors. Thereby blocking the expression of inflammatory target genes which contribute to the invasive potential of cancer cells. In this study we investigated the effects of inhibiting KPNB1 using siRNA and a novel small molecule, INI-43, on cancer cell migration and invasion as well as NFkB and AP-1 transcriptional function.

## RESULTS

### KPNB1 is required for the migration and invasion of cervical cancer cells

**Figure 1:**
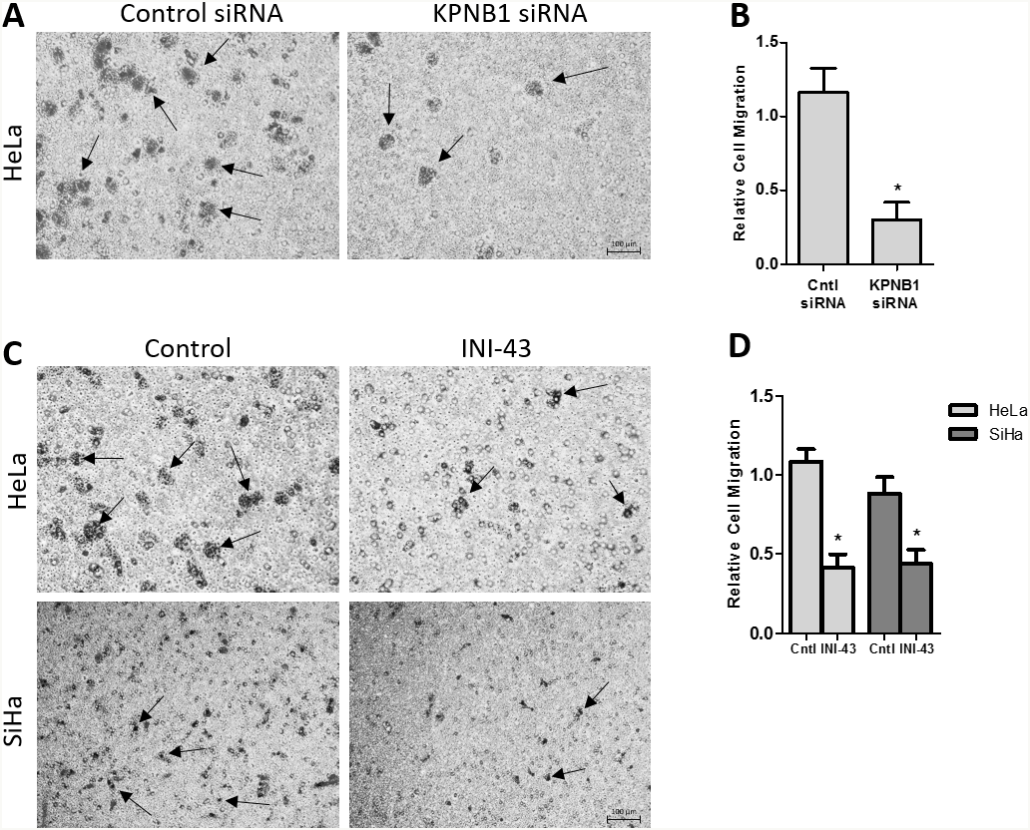
Effect of KPNB1 inhibition on cervical cancer cell migration **(A)** Representative images from the transwell migration chamber showing migration of HeLa cells following KPNB1 knockdown, arrows identifying migrating cells. Scale bar represents 100 μm. **(B)** Quantification of transwell migration assay following KPNB1 knockdown normalized to MTT cell viability. **(C)** Representative images showing HeLa and SiHa cell migration through the membrane following a 3 hour 10 μM INI-43 pre-treatment, arrows identifying migrating cells **(D)** The number of HeLa and SiHa cells that migrated through the transwell chamber following INI-43 treatment were quantified and normalized to MTT cell viability. Results shown are the mean ± SD of experiments performed in triplicate. (*p<0.05).

**Figure 2:**
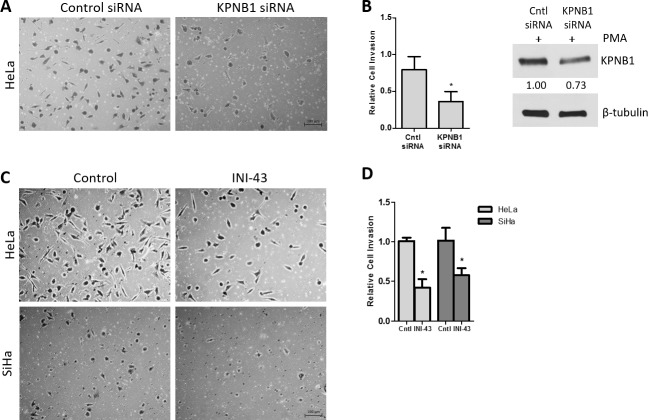
Effect of KPNB1 inhibition on cervical cancer cell invasion **(A)** Representative images of the invasion transwell assay showing invasion of HeLa cells through the matrigel-coated transwell membrane following KPNB1 knockdown. Scale bar represents 100 μm. **(B)** Quantification of the invasion assay following KPNB1 knockdown normalized to MTT cell viability. Western blot confirmed KPNB1 knockdown. **(C)** Representative images of the invasion assay following 3 hr 10 μM INI-43 pre-treatment of HeLa and SiHa cells showing the number of cells able to invade the matrigel-coated membrane following nuclear import inhibition. **(D)** Quantification of invasion assay following INI-43 treatment, normalized to MTT cell viability. Results shown are the mean ± SD of experiments performed in triplicate. (*p<0.05).

**Figure 3:**
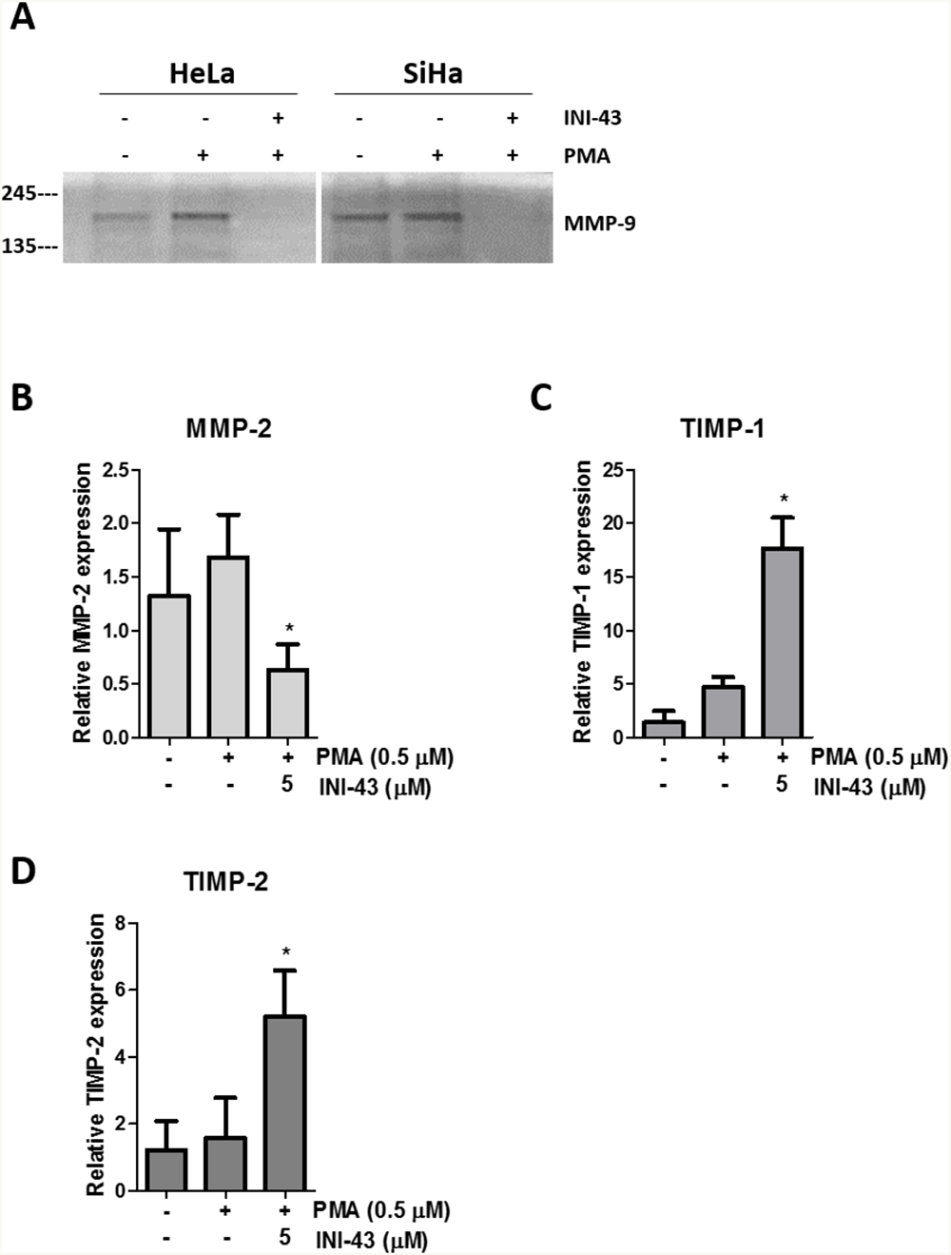
KPNB1 inhibition affects MMP-9 activity and MMP-2, TIMP-1 and TIMP-2 expression **(A)** Gelatin Zymography showing MMP-9 activity in HeLa and SiHa conditioned media collected after 16 hours following a 3 hour pre-treatment of 10 μM INI-43 and a 1 hour 0.5 μM PMA stimulation. qRT-PCR analysis of mRNA expression following 5 μM INI-43 treatment for 21 hours with the addition of 0.5 μM PMA for a further 3 hours in HeLa cells shown for MMP-2 **(B)** and inhibitors of matrix metalloproteases, TIMP-1 **(C)** and TIMP-2 **(D)**. Results shown are the mean ± SD of experiments performed in triplicate. (*p<0.05).

In our previous studies we have reported that KPNB1 is required for the survival and proliferation of cervical cancer cells and that inhibition of its expression and activity resulted in cell death via apoptosis [5, 37]. Little is known about the role of KPNB1 in other cancer phenotypes such as migration and invasion. In this study, we investigated the requirement of KPNB1 expression and activity for cancer cell migration and invasion. Using transwell migration assays, we showed that knocking down KPNB1 using siRNA reduced the ability of HeLa cervical cancer cells to migrate through the transwell membrane in the presence of PMA stimulation (Figure 1A). Quantification of the migration of control and KPNB1 siRNA treated cells shows a significant reduction in migration when KPNB1 expression is inhibited (Figure 1B). The small molecule inhibitor of nuclear import, INI-43, was also able to reduce the migratory ability of HeLa and SiHa cervical cancer cells (Figure 1C). Quantification of migratory ability following INI-43 treatment shows significant reductions in migration in both cervical cancer cell lines (Figure 1D). The invasive ability of cervical cancer cells was assessed using a transwell invasion plate with matrigel-coated chambers. The inhibition of KPNB1 by both siRNA (Figure 2A & 2B) and INI-43 (Figure 2C & 2D) significantly interfered with both HeLa and SiHa cancer cell invasion. As these assays assessed the role of nuclear import inhibition of cancer cell motility, the effect of cell death was eliminated by optimizing treatment duration and normalizing the results to concurrent live cells as measured using the MTT cell proliferation assay. The ability of cancer cells to invade the extracellular matrix is dependent on the activity of matrix metalloproteases (MMPs). We therefore assessed gelatinase activity of MMP-9 using a gelatin Zymography and looked at MMP-2, TIMP-1 and TIMP-2 expression using qRT-PCR following nuclear import inhibition. Conditioned media was collected from HeLa and SiHa cells following INI-43 treatment and PMA stimulation and the results showed a substantial reduction in MMP-9 activity following nuclear import inhibition (Figure 3A). The expression of MMP-2 was found to be downregulated following INI-43 treatment in HeLa cells (Figure 3B) while the inhibitors of matrix metalloproteases, TIMP-1 and TIMP-2, were significantly upregulated (Figure 3C & 3D). Together these results provide further evidence for the role of KPNB1 in cancer cell biological processes such as migration and invasion.

### Inhibition of KPNB1 interferes with NFkB subcellular localisation

**Figure 4:**
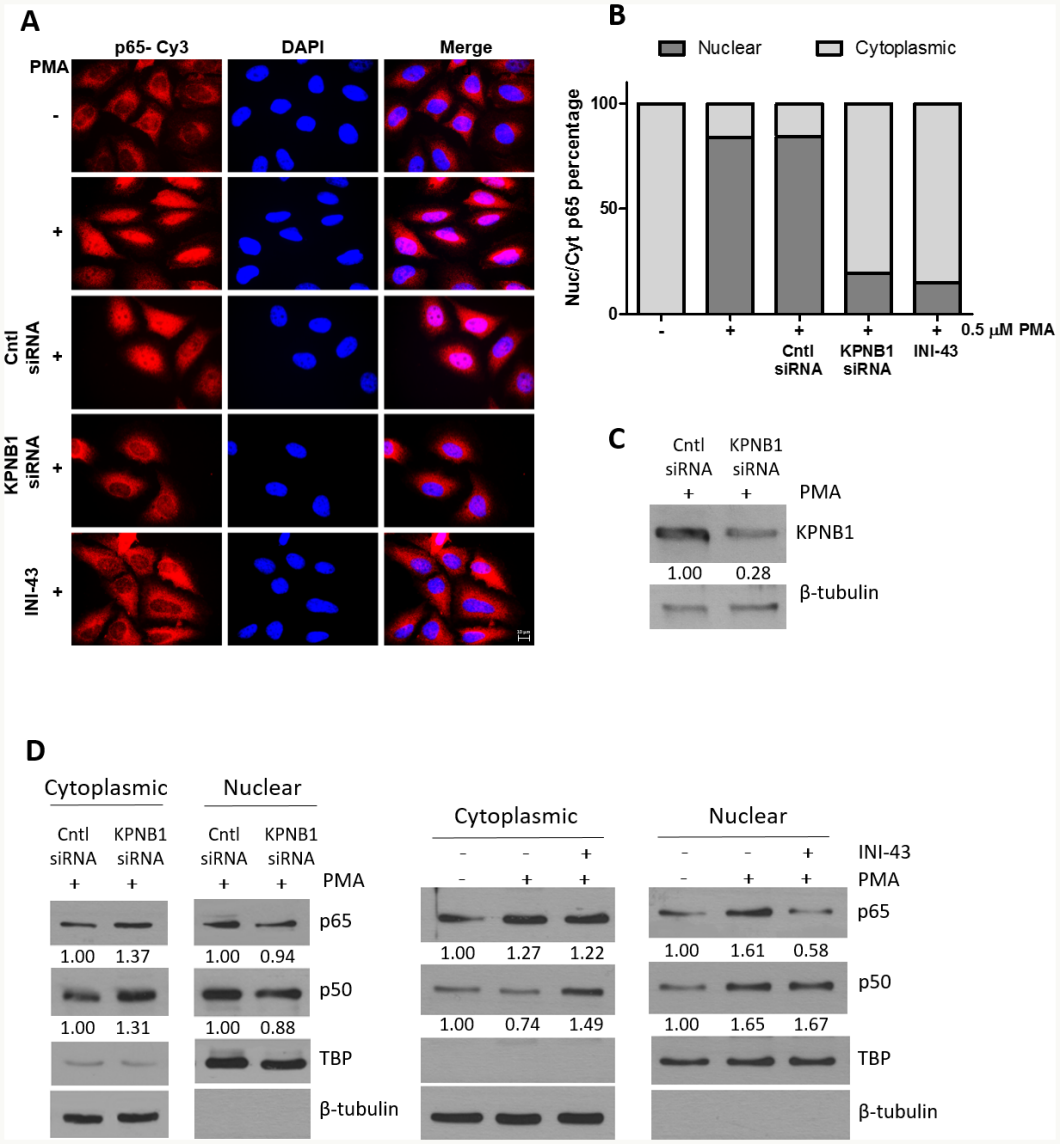
NFkB localisation following KPNB1 inhibition **(A)** Representative immunofluorescent images showing NFĸB p65 expression and localisation as well as nuclear staining (DAPI) in HeLa cells. The results show the changes in cellular localisation of NFkB following a 1 hour treatment with 0.5 μM PMA, as well as inhibiting KPNB1 through siRNA knockdown or 3 hour pre-treatment with 10 μM INI-43 in addition to PMA stimulation (x100 Objective). Scale bar represents 10 μm. **(B)** Screening of 250 cells over 25 fluorescent images per treatment were scored into “predominantly nuclear” or “predominantly cytoplasmic” p65 fluorescence according to nuclear/cytoplasmic fluorescent intensity analysis. The graph was plotted using the percentage nuclear and cytoplasmic p65 fluorescence. **(C)** Knockdown of KPNB1 was confirmed by western blot. **(D)** Western blot analysis of NFĸB p65 and p50 expression in the cytoplasmic and nuclear protein fraction of HeLa cells transfected with KPNB1 siRNA or pre-treated with 10 μM INI-43 for 3 hours before a 1 hour 0.5 μM stimulation with PMA. β-tubulin was used as a cytoplasmic loading control while TBP (TATA-binding protein) was used as a nuclear loading control. Densitometric analysis was normalized to loading controls.

**Figure 5:**
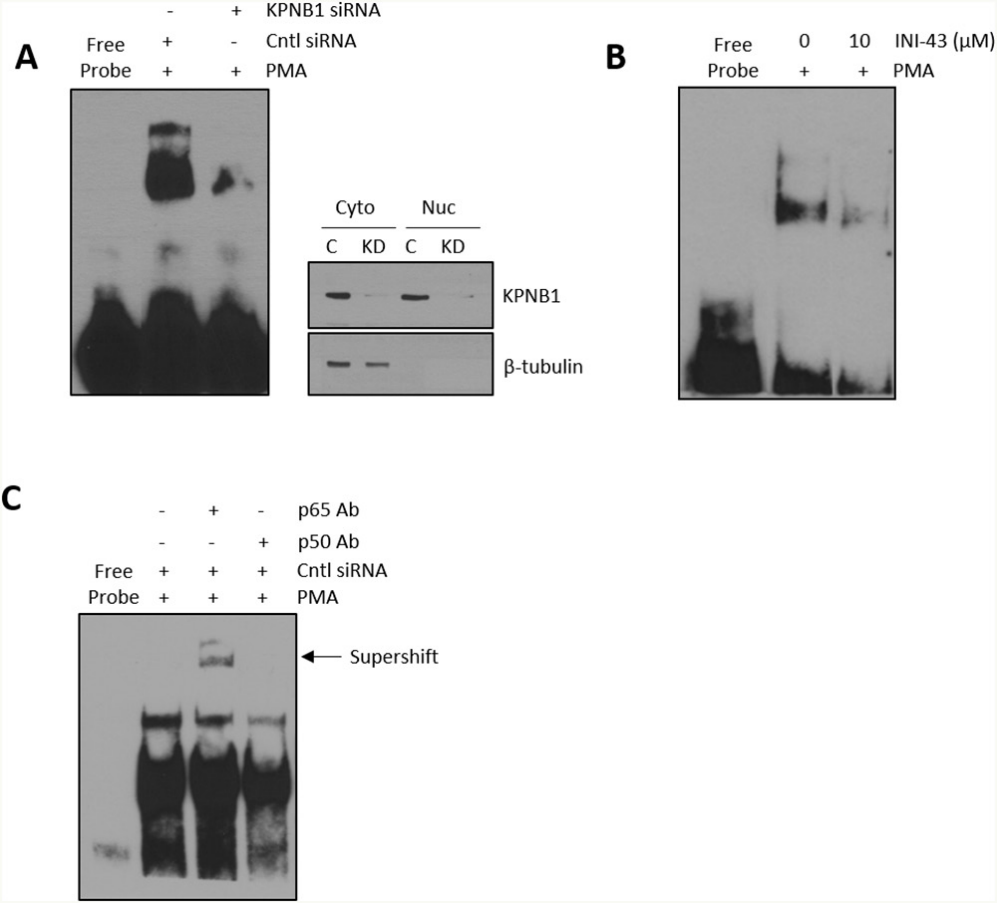
Formation of a nuclear NFkB/DNA binding complex following KPNB1 inhibition **(A)** Electromobility shift assay (EMSA) showing binding of nuclear protein to a biotin-labelled NFĸB oligonucleotide following KPNB1 siRNA transfection in PMA-stimulated HeLa cells. Western blot analysis of fractionated protein samples confirmed KPNB1 knockdown and fractionation. **(B)** EMSA of nuclear extracts obtained from cells pre-treated with 10 μM INI-43 for 2 hours followed by a 1 hour 0.5 μM PMA stimulation. **(C)** Supershift analysis with the NFĸB p65 antibody showing a shift in the DNA-protein complex while the p50 antibody reduced protein binding both confirming the presence of NFkB in the DNA-protein complex.

Cancer cell proliferation, migration and invasion has a strong association with the correct function of transcription factors that associate with cancer such as NFkB. For NFkB to be active as a transcription factor it is essential that it is transported into the nucleus where it can bind the DNA and initiate transcription of its target genes. Here, we investigated the requirement of the nuclear import protein, KPNB1, for subcellular localisation of the NFkB p65/p50 dimer in cervical cancer cells using multiple techniques including; immunofluorescent analysis, nuclear/cytoplasmic protein separation and electromobility shift assays (EMSA). Translocation of NFkB into the nucleus was stimulated using PMA. Immunofluorescent analysis shows that PMA treatment results in the translocation of NFkB into the nucleus but inhibiting KPNB1 through siRNA transfection or INI-43 pre-treatment blocks nuclear translocation of NFkB and causes its cytoplasmic retention (Figure 4A). Quantification of fluorescent images shows an overall reduction in nuclear NFkB in KPNB1 inhibited cells following PMA stimulation (Figure 4B). Western blotting was used to validate KPNB1 knockdown (Figure 4C). These findings were independently validated using nuclear/cytoplasmic fractionation of protein samples followed by western blot analysis for the p65 and p50 subunits of NFkB. In the presence of KPNB1 siRNA or treatment with INI-43, a decrease in nuclear and concomitant increase in cytoplasmic NFkB p65 and p50 was observed (Figure 4D). To further elucidate the effect of inhibiting KPNB1 on NFkB, EMSAs were performed to investigate the ability of nuclear NFkB to bind a NFkB consensus sequence. A substantial decrease in the presence of the nuclear NFkB/DNA complex was observed following nuclear import inhibition using KPNB1 siRNA or INI-43 treatment (Figure 5A & 5B). Western blotting was used to confirm protein nuclear/cytoplasmic fractionation and KPNB1 knockdown (Figure 5A). Supershift analysis with antibodies specific to the NFkB p65 and p50 subunits identified NFkB p65 as a protein present in the complex. A reduction in the binding when the supershift was performed using the NFkB p50 antibody suggests competitive binding of the protein with the biotin-labelled oligonucleotide (Figure 5C). Together, these results confirm that KPNB1 is necessary for NFkB nuclear localisation.

### NFkB transcriptional activity and inflammatory target gene expression are inhibited in KPNB1-inhibited cells

**Figure 6:**
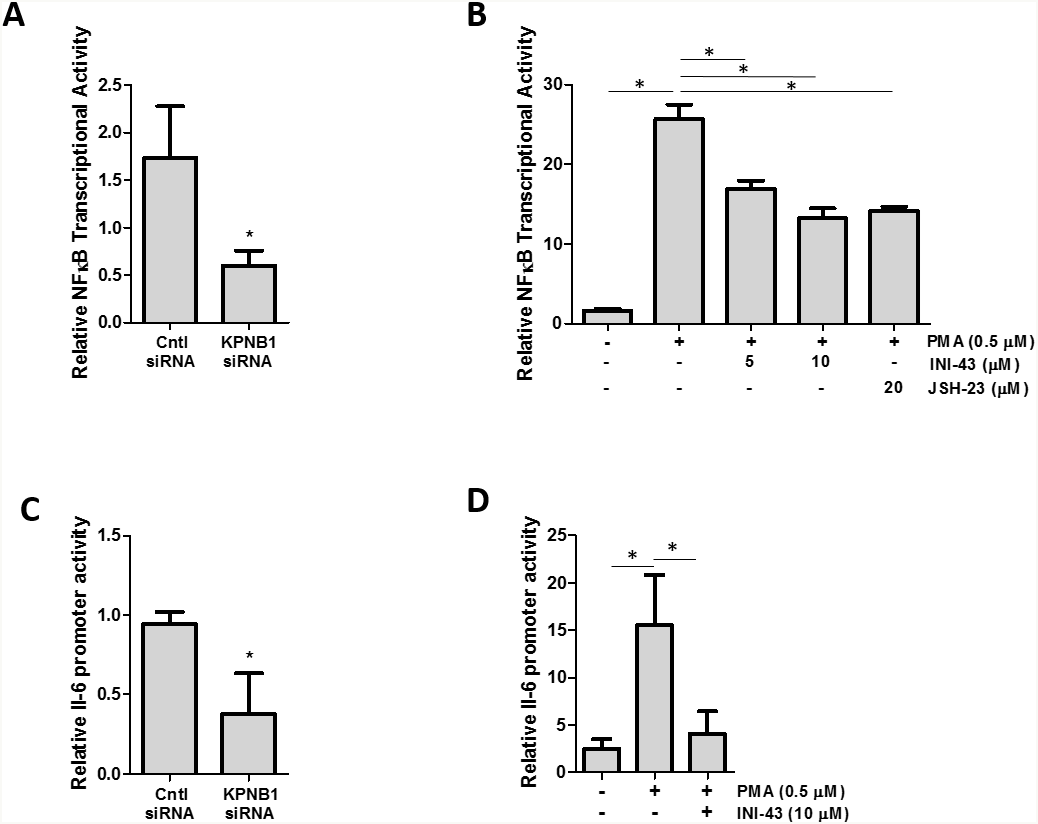
KPNB1 inhibition affects NFkB transcriptional activity **(A)** NFĸB transcriptional activity using an NFkB binding site-luciferase reporter transiently transfected into HeLa cells following KPNB1 knockdown and stimulation with PMA. **(B)** NFĸB transcriptional activity in HeLa cells following a 3 hour 0.5 μM PMA stimulation but subsequently inhibited following a 21 hour pre-treatment with INI-43 and JSH-23 treatment (24 hr total treatment time). **(C)** IL-6 promoter activity shown following KPNB1 knockdown and stimulation with PMA. **(D)** The effect of 0.5 μM PMA stimulation for 3 hours on IL-6 promoter activity is shown in HeLa cells followed by pre-treatment with 10 μM INI-43 for 21 hours. Results shown are the mean ± SD of experiments performed in quadruplicate. (*p<0.05)

**Figure 7:**
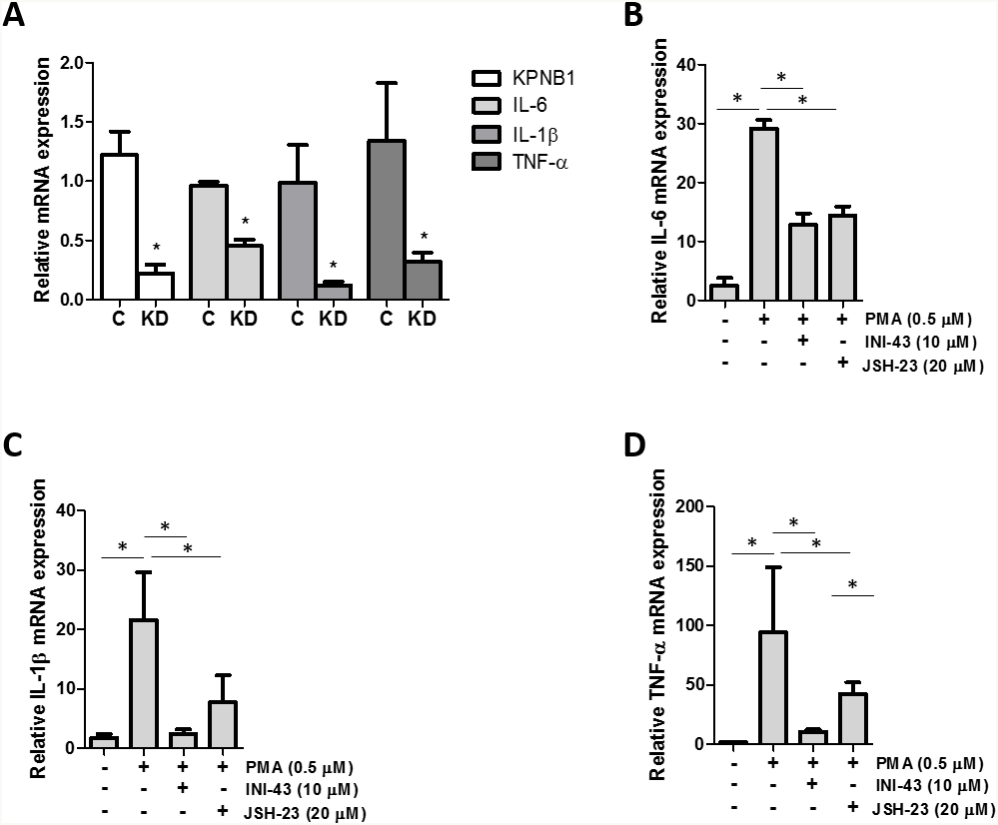
KPNB1 inhibition affects inflammatory NFkB target gene expression **(A)** qRT-PCR analysis of mRNA expression showed the effect of KPNB1 knockdown (C= Control, KD= Knockdown) on KPNB1 mRNA expression, confirming knockdown, as well as expression of inflammatory target genes; IL-6, IL-1β and TNF-α. **(B)** IL-6 mRNA expression shown following stimulation for 1 hour with 0.5 μM PMA as well as pre-treatment for 2 hours with 10 μM INI-43 or 23 hours 20 μM JSH-23 followed by 1 hour PMA stimulation. The same conditions were used to look at IL-1β mRNA expression **(C)** and TNF-α **(D)**. Target gene expression was normalized to expression of the housekeeping gene, GAPDH. Results shown are the mean ± SD of experiments performed in triplicate. (*p<0.05)

Since it was established that NFkB requires KPNB1 for its transport into the nucleus, we postulated that inhibiting KPNB1 should result in a change in NFkB transcriptional activity and NFkB target gene expression. The transcriptional activity of NFkB was quantified using a luciferase reporter assay of both an artificial NFkB consensus binding site containing promoter as well as a known NFkB target gene, the IL-6 promoter-luciferase construct. Our data shows that inhibiting KPNB1 using siRNA resulted in significantly reduced transcriptional activity of the NFkB promoter-luciferase construct (Figure 6A). Treatment of HeLa cells with INI-43 also resulted in a significant reduction of PMA-stimulated NFkB transcriptional activity in a dose-dependent manner to a level which was comparable to the effect of the NFkB inhibitor, JSH-23 (Figure 6B). The IL-6 promoter was similarly inhibited by both KPNB1 siRNA (Figure 6C) and INI-43 treatment (Figure 6D). As inhibiting KPNB1 resulted in an inhibition of NFkB activity we next investigated whether inhibiting nuclear import affects the expression of NFkB target genes specifically involved in inflammatory signaling associated with cell biology changes in cancer. Inflammatory cytokines; IL-6, IL-1β and TNF-α are target genes of NFkB and the expression was monitored in response to KPNB1 inhibition. HeLa cells transfected with KPNB1 siRNA showed not only reduced mRNA expression of the KPNB1 gene but a significant reduction in the mRNA expression of all three inflammatory cytokines (Figure 7A). The small molecule inhibitor, INI-43, had a similar inhibitory effect and significantly inhibited PMA-stimulated IL-6 (Figure 7B), IL-1β (Figure 7C) and TNF-α (Figure 7D) gene expression which was comparable to the effect of the NFkB inhibitor, JSH-23. The enhanced reduction of TNF-α mRNA expression in INI-43 treated cells in comparison to the effect of JSH-23 suggests that KPNB1 inhibition by INI-43 on NFkB alone may not be fully responsible for the effect of INI-43 on inflammatory target gene expression (Figure 7D). Other transcription factors such as AP-1 are also known to regulate cytokine expression, namely IL-6, and may too depend on KPNB1 for its activity. Together these results provide evidence that the inhibition of nuclear import via KPNB1 associates with decreased expression of pro-inflammatory cytokine expression.

### KPNB1 inhibition affects activity and target gene expression of the AP-1 transcription factor

**Figure 8:**
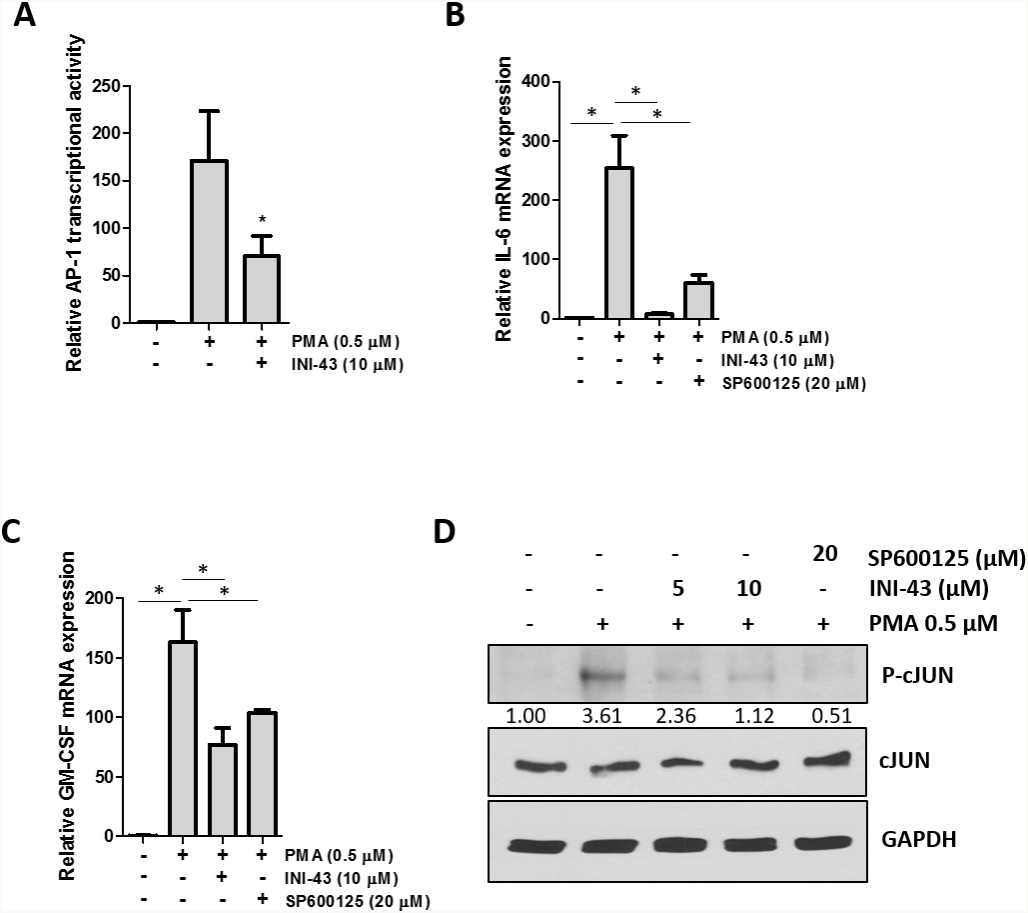
AP-1 activity and target gene expression in affected by KPNB1 inhibition **(A)** A luciferase reporter assay showed AP-1 transcriptional activity in HeLa cells following a 3 hour 0.5 μM PMA treatment as well as a 21 hour pre-treatment with 10 μM INI-43. **(B)** qRT-PCR analysis of AP-1 target genes IL-6 and **(C)** GM-CSF shows mRNA expression following stimulation for 1 hour with 0.5 μM PMA and a 2 hour pre-treatment with 10 μM INI-43 or 1 hour pre-treatment with 20 μM SP600125. **(D)** Western blotting showed phosphorylation levels of c-JUN in HeLa cells following a 1 hour treatment with PMA and a 2 hour pre-treatment with INI-43 or 1 hour pre-treatment with SP600125. Densitometric analysis shows phosphorylated levels of c-JUN normalized to total c-JUN. Results shown are the mean ± SD of experiments performed in triplicate. (*p<0.05)

Transcription factors, other than NFkB, also contain a nuclear localisation sequence (NLS) and may depend on KPNB1 for nuclear entry. The transcription factor AP-1 has been reported to have a NLS present on the c-JUN component, although nuclear import of c-JUN is not limited to KPNB1 only [40]. We investigated whether KPNB1 may also influence activity and target gene expression of the AP-1 transcription factor. The transcriptional activity of AP-1 was quantified using a luciferase reporter construct containing multiple artificial AP-1 binding sites. Our data shows that the small molecule inhibitor of nuclear import, INI-43, was able to significantly reduce PMA-stimulated AP-1 transcriptional activity (Figure 8A). The expression of inflammatory-associated target genes of AP-1; IL-6 (Figure 8B) and GM-CSF (Figure 8C) were analysed using qRT-PCR. Stimulation with PMA significantly increased mRNA expression of both genes while pre-treatment with INI-43 was able to reduce this expression which was comparable to the effect of the JNK inhibitor, SP600125, which is an upstream inhibitor of the AP-1 signaling pathway. Activation of AP-1 requires that c-JUN be phosphorylated in the nucleus by phosphorylated JNK, although nuclear import of JNK is NLS-independent and therefore not reliant on KPNB1 [41, 42]. Western blotting was used to assess the phosphorylation of c-JUN and showed that PMA stimulation increased c-JUN phosphorylation while INI-43 treatment at 0.5x and 1x IC_50_ as well as SP600125 reduced phosphorylation (Figure 8D). These results show that the AP-1 transcription factor is dependent on KPNB1 for its activity and target gene expression.

### Cervical cancer cell motility depends on inflammatory transcription factor activity

**Figure 9:**
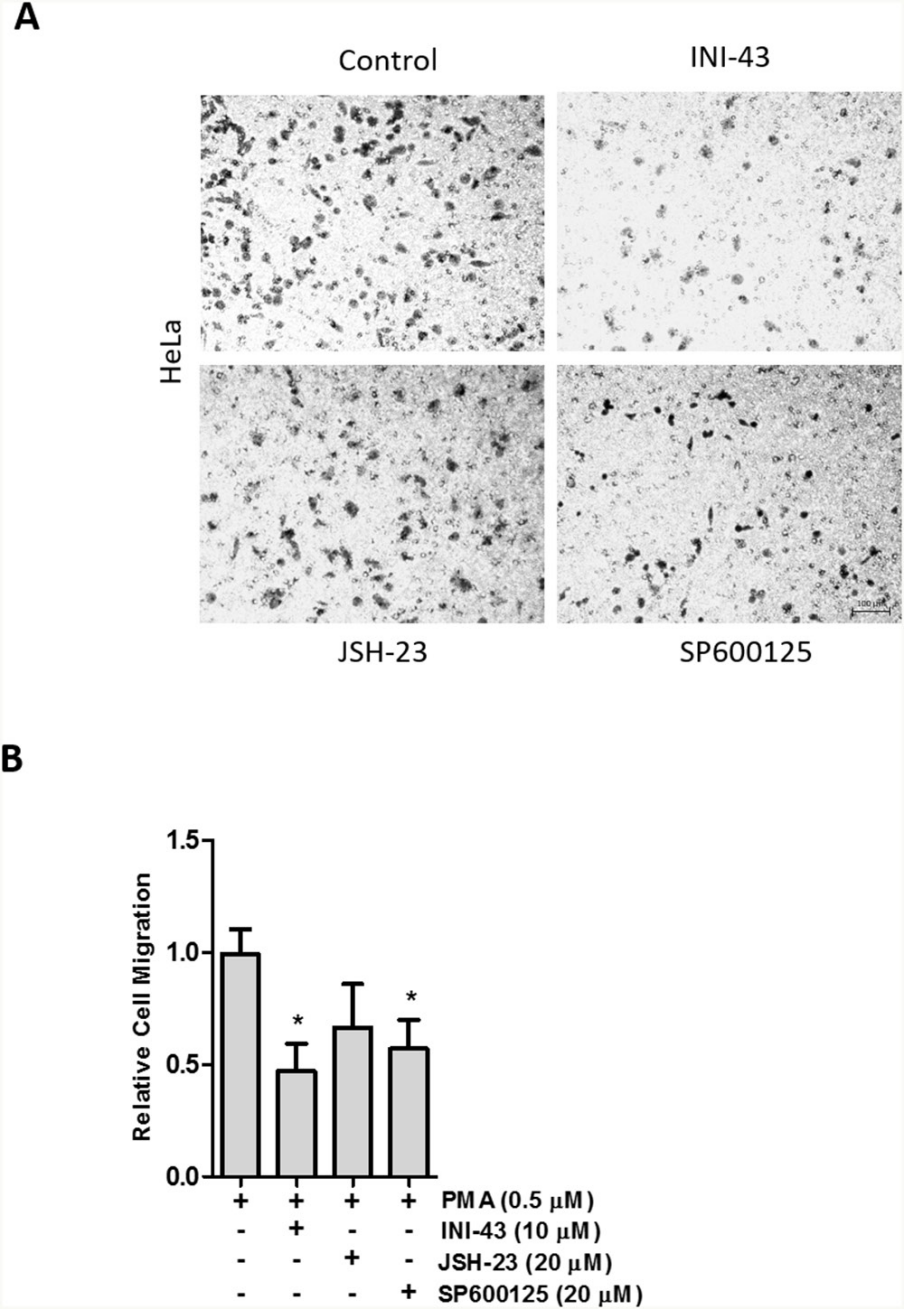
Cervical cancer cell migration depends on AP-1 signaling activity **(A)** Representative images of a transwell migration assay showing the amount of HeLa cells that were able to migrate through the membrane in the presence of 0.5 μM PMA (control) and following a 3 hour pre-treatment with 10 μM INI-43 and 20 μM JSH-23 and a 2 hour pre-treatment with 20 μM SP600125. Scale bar represents 100 μm. **(B)** Quantification of the migration assay showing HeLa cell migration following INI-43, JSH-23 and SP600125 treatment, normalized to MTT cell viability. Results shown are the mean ± SD of experiments performed in triplicate. (*p<0.05)

There is evidence in the literature that NFkB and AP-1 signaling contributes to cancer cell migration and invasion through the expression of inflammatory cytokines such as IL-6 and TNF-α [43]. We have shown above that NFkB and AP-1 signaling can be modulated through inhibiting the function of KPNB1. As a result of this we wanted to investigate whether the changes is cervical cancer cell motility seen when KPNB1 was inhibited are potentially attributed to the inhibition of NFkB and AP-1 signaling. In order to do this we performed a transwell migration assay where HeLa cells were treated with INI-43 alongside cells where NFkB was inhibited (JSH-23) as well as AP-1 signaling inhibited (SP600125). Although inhibiting NFkB signaling showed a trend towards reducing HeLa cancer cell migration the results were not significantly different from the control while inhibiting KPNB1 using INI-43 and inhibiting AP-1 signaling using SP600125 significantly reduced the migratory ability of cervical cancer cells (Figure 9A). Quantification of the migration data is shown in Figure 9B. The data suggests that suppressed NFkB and AP-1 signaling following KPNB1 inhibition may together contribute to the inhibitory effects on cancer cell biology.

## DISCUSSION

The increased expression of KPNB1 has been shown to increase nuclear import efficiency in transformed cells [44]. This enhanced access of cargo into the nucleus is suggested to sustain the high metabolic and proliferative demands of cancer cells. Cancer cells have been found to require KPNB1 for their proliferation although the exact mechanism of action for this requirement is not fully understood. It could be attributed to either; the essential role of KPNB1 in mitosis or linked to the inability of certain cargoes to gain access into the nucleus and perform their jobs in promoting proliferation [45]. This led us to question whether other cancer phenotypes are also dependent on KPNB1.

In our study we have shown that inhibiting KPNB1 reduced migration and invasion of cancer cells. As cancer cells require the activity of matrix-modifying enzymes to invade surrounding tissue, we looked at whether MMP activity was affected by KPNB1 inhibition. MMP activity was found to be significantly inhibited in cervical cancer cells following INI-43 treatment. Fan *et al*. (2016) found MMP-9 to be upregulated in cervical cancer tissue which correlated with a poor patient prognosis. Inhibiting MMP-9 activity in SiHa and HeLa cervical cancer cells reduced their migratory and invasive ability [46]. Similarly, MMP-2 expression was also found to be decreased following KPNB1 inhibition while expression of the inhibitors of matrix metalloproteases, TIMP-1 and TIMP-2, were significantly increased. TIMPs are known to be downregulated in a variety of cancer cell lines which is thought to contribute to the invasive properties of cancer cells. The overexpression of TIMP-2 in a highly metastatic melanoma cell line was able to inhibit metastasis [47, 48]. This work suggests that KPNB1 may play an important role in the migratory and invasive potential of cervical cancer cells. KPNB1 could be functioning as one of the key transporters of transcription factors associated with cancer cell migration, invasion and metastasis.

The transcription factors NFkB and AP-1 play a primary role in regulating MMP expression [14, 29, 49]. As both NFkB and AP-1 require access to the nucleus in order to be functional we hypothesized that inhibiting nuclear import via KPNB1 may lead to their inactivity and ultimately various downstream effects on cancer cell biology. We identified KPNB1 to be required for the subcellular translocation and activity of the NFkB transcription factor. A study by Liang *et al*. (2013) found KPNB1 to be responsible for the cellular translocation of NFkB p65 and inhibiting its function resulted in the retention of NFkB p65 in the cytoplasm leading to reduced NFkB transcriptional activity [23]. Our data is in support of these findings. NFkB is an important mediator of inflammation while other transcription factors such as AP-1 have also been known to initiate expression of inflammatory cytokines [26, 33]. In this study we questioned whether inhibiting NFkB and AP-1 activity via inhibiting KPNB1 associated with the inhibition of inflammatory gene expression. KPNB1 was inhibited using two approaches; RNA interference and drug-mediated inhibition. The canonical NFkB pathway as well as c-JUN activation pathway was stimulated in cell culture using PMA which enhanced IL-1β, IL-6, TNF-α and GM-CSF expression. We show that stimulated cytokine expression could be blocked following inhibition of KPNB1 or through transcription factor specific inhibitors further confirming the reliance of NFkB and AP-1 activity on the nuclear importer. The pro-inflammatory cytokines; IL-1β, IL-6, TNF-α and GM-CSF have been implicated in cell biology changes required for tumour progression [11, 50–52]. These changes include increased migratory ability and invasive potential which are required for the epithelial-to-mesenchymal transition (EMT) that precedes metastasis [53]. On activation of the transcription factor, NFkB, target genes such as TNF-α and IL-6 can subsequently form a positive feedback loop further activating NFkB as well as other transcription factors such as AP-1. Together activation of these transcription factors has been associated with down-regulated E-cadherin expression and upregulation of matrix metalloprotease production ultimately promoting EMT [29, 54, 55].

To identify whether either NFkB or AP-1 are required for the migration of cervical cancer cells, each transcription factor was individually inhibited alongside INI-43 and the effect on migration analysed. Although inhibition of the AP-1 signaling pathway was able to significantly reduce migration, neither inhibition of AP-1 or NFkB on their own were able to reduce migratory potential of cancer cells to the extent of the nuclear import inhibitor. Transcription factors of the FOS/JUN, CREB/ATF and NFkB families are known to act synergistically to significantly increase the expression of the same target gene. It has therefore been proposed that inhibiting more than one transcription factor acting synergistically may be a more appropriate approach [56]. Darnell (2002) proposed that inhibiting nuclear import proteins may be a way of specifically targeting multiple overactive transcription factors [57]. Our data provides evidence that supports Darnell's proposal, showing that inhibiting KPNB1 affects AP-1 and NFkB transcriptional activities required for cancer cell biology.

To our knowledge this study is a first to identify KPNB1 as a potential therapeutic target for inflammatory signaling in cancer associated with enhanced motility and invasiveness. Much research has been done on targeting individual transcription factors as a chemotherapeutic approach. This approach is often associated with broad-range side effects given the diverse role of each transcription factor in normal cellular functioning. Cancer cells however, have been reported to become “addicted” and highly dependent on the activity of certain transcription factors or oncogenes, hence inhibiting their function is thought to effect cancer cells to a greater extent than non-cancer cells [58]. Ideally a targeted approach would be required to limit such off-target effects. Targeting KPNB1 as a means of transcription factor inhibition may be a favorable approach as KPNB1 is overexpressed in cervical cancer tissue in comparison to normal cervical tissue and we have previously shown that normal cells are less dependent on KPNB1 for proliferation and survival [5]. Our research group has also previously shown that inhibiting KPNB1 using INI-43 reduced tumour growth in a mouse model [37]. These results further characterize the nuclear import protein, KPNB1, as a potential anticancer target.

## MATERIALS AND METHODS

### Cell culture

Human cervical carcinoma cell line, HeLa and SiHa, were obtained from the American Type Culture Collection (ATCC). Cells were cultured under adherent conditions in Dulbecco's Modified Eagle's Medium (DMEM), supplemented with penicillin (100 U/ml), streptomycin (100 μg/ml) and 10% Fetal Calf Serum (FCS) (HiClone, Thermo Scientific, USA). Cells were incubated at 37°C in 95% air and 5% CO_2_. Cell lines were authenticated by DNA profiling using the Cell ID system (Promega, USA).

### RNA interference & drug treatment

Short-interfering RNA (siRNA) was used to inhibit KPNB1 gene expression (sc-35736, Santa Cruz Biotechnology, USA). Control siRNA (SIC001, Sigma-Aldrich, USA) consisting of a scrambled RNA sequence was used as a non-silencing control. The cells were transiently transfected with 20 nM (35 mm plate)/ 52 nM (60 mm plate) siRNA using TransFectin Lipid Reagent (Bio-Rad, USA). Protein was extracted or cells assayed 30-48 hrs post transfection. The effect of KPNB1 knockdown was confirmed by Western Blot analysis. The nuclear import inhibitor, INI-43, was used at a concentration of 10 μM unless otherwise stated [37]. Other inhibitors include the NFkB inhibitor, JSH-23, used at 20 μM (Sigma-Aldrich, USA) and the JNK inhibitor, SP600125, used at 20 μM (Sigma-Aldrich, USA). The phorbol ester, phorbol-12-myristate-13-acetate (PMA) (Sigma-Aldrich, USA), was used to stimulate a cellular inflammatory response.

### Migration and invasion assays

Cells were seeded into 35 mm dishes and treated accordingly before being trypsinized, resuspended in 0.1% FCS-containing DMEM and seeded in equal quantities into 12-well Transwell migration chambers (Greiner Bio-One, Austria) or 24-well matrigel covered Transwell invasion chambers (BD Biosciences, USA) with an 8 μm pore size. The chambers were placed into a lower chamber containing 20% FCS-containing DMEM and the cells allowed to migrate/invade through the membrane or matrigel matrix over 24 hrs. The cells that were unable to move through the membrane were removed while the remaining cells were fixed in methanol, stained with crystal violet, counted and imaged using a Zeiss Primovert inverted phase microscope. Results were normalized to an MTT cell viability assay (according to the manufacturer's protocol, Sigma-Aldrich, USA) and western blotting confirmed KPNB1 knockdown.

### Gelatin zymography

Cells were seeded into 35 mm plates, treated and serum-free media placed onto the cells to condition for 16 hrs. Conditioned media was collected and centrifuged to remove any cellular debris, combined with sample buffer, loaded into a gelatin gel and run at 125 V. The gel was removed and placed in a renaturing solution before being placed in the developing buffer and allowed to incubate at 37°C overnight to facilitate gelatinase activity. The staining solution was used to dye the gel while the destaining solution exposed areas of gelatinase activity.

### Immunofluorescent microscopy

Immunofluorescent analysis of NFĸB p65 was performed on HeLa cells plated over glass coverslips and transfected with siRNA (48 hrs), treated with 10 μM INI-43 (3 hrs) and/or stimulated with 0.5 μM PMA for 1 hour before being fixed in 4% paraformaldehyde. Cells on the coverslips were permeabilised using 0.25% Triton X-100 in PBS for 10 mins followed by three 5 min PBS washes. Cells were blocked in 1% BSA in PBS-T + 0.3 M Glycine (for quenching) at room temperature for 30 mins. Cells were subsequently incubated with α-NFĸB p65 primary antibody (1:200; sc-7151x, Santa Cruz Biotechnology, USA) in 1% BSA in PBS-T for 1 hour at room temperature, followed by three 5 min PBS washes. Cy3-conjugated goat anti-rabbit secondary antibody (1:300, Jackson ImmunoResearch, USA) in 1% BSA in PBS-T was applied for a further 1 hour. Cells were washed three times for 5 mins in PBS, nuclei stained with DAPI (100 ng/ml) and mounted onto glass slides with Mowiol. Fluorescent images were captured at 100x in oil immersion using a Zeiss Axiovert 200M fluorescent microscope with AxioVision 4.8 Zeiss software and an AxioCam HRm.

### Protein harvest and western blot analysis

Following relevant treatment of cells, protein was either harvested on ice in RIPA buffer containing a fresh mixture of complete protease inhibitors (Roche, Switzerland) and 0.1 M Sodium Orthovanadate to inhibit phosphatase activity or fractionated using the NE-PER Nuclear and Cytoplasmic Extraction Reagents according to the manufacturer's instructions (Thermo Scientific, USA). Protein concentrations were determined using the Bicinchoninic Acid (BCA) assay kit (Pierce, Thermo Scientific, USA). Western blot analysis was performed using rabbit anti-KPNB1 (H-300) (sc-11367, Santa Cruz Biotechnology, USA), rabbit anti-NFĸB p65 (H-286) (sc-7151x, Santa Cruz Biotechnology, USA), rabbit anti-NFĸB p50 (H-119) (sc-7178x, Santa Cruz Biotechnology, USA), rabbit anti-p-c-Jun (Ser63/73) (sc-16312-R, Santa Cruz Biotechnology, USA), rabbit anti-c-Jun (D) (sc-44, Santa Cruz Biotechnology, USA), mouse anti-GAPDH (0411) (sc-47724, Santa Cruz Biotechnology, USA), rabbit anti-β-tubulin (H-235) (sc-9104, Santa Cruz Biotechnology, USA) and rabbit anti-TBP (N-12) (sc-204, Santa Cruz Biotechnology, USA).

### Electromobility shift assay (EMSA) and supershift assay

Nuclear protein was extracted from treated HeLa cells and concentrations quantified as before. The wild-type NFkB oligonucleotides; 5′-AGTTGAGGGGACTTTCCCAGGC-3′ and 5′-TCA ACTCCCCTGAAAGGGTCCG-3′ were labelled using the Biotin 3′ End Labelling Kit (Thermo Scientific, USA) according to the manufacturer's instructions. Equal parts of labelled probe were annealed under the following conditions; 3 mins- 95°C, 10 mins- 65°C, 60 mins- 37°C and 1 min- 25°C. The binding reaction contained; 5 μg protein, incubation buffer, poly DI/DC and 5 μl biotin-labelled double-stranded NFĸB oligonucleotide. For the supershift analysis, 2 μl NFĸB p65/p50 antibody was added to the binding reaction. For the controls 1 μl 50 μM unlabeled double-stranded wild-type oligonucleotide or unlabeled double-stranded mutant oligonucleotide, 5′-AGTTGAGGCGACTTTCCCAGGC-3′ and 5′-GCC TGGGAAAGTCGCCTCAACT-3′, was added to the binding reaction. Samples were electrophoresed on a polyacrylamide gel and detected using the Chemiluminescent Nucleic Acid Detection Module (Thermo Scientific, USA).

### Luciferase reporter assays

HeLa cells were cultured in 24-well plates and transfected with 50 ng of the NFĸB p65 luciferase reporter construct (containing 5 copies of the p65 binding site, Promega, USA), the AP-1 luciferase reporter construct (containing four copies of the AP-1 binding site, [28]) or the full-length IL-6 promoter construct in the pXP2 luciferase vector (Gifted by Assoc Prof Luiz Zerbini, ICGEB, Cape Town) [59] and 5 ng of the pRL-TK plasmid (encoding Renilla luciferase) to normalize for transfection efficiency. Thereafter cells were transfected with siRNA (48 hrs), treated with 10 μM INI-43 (3-24 hrs), 20 μM JSH-23 (24 hrs) and/or stimulated with 0.5 μM PMA for 1-8 hours before being lysed in 100 μl 1x Passive Lysis Buffer (Promega, USA). Luciferase firefly activity was assayed using the Dual Luciferase kit (Promega, USA) on the Glomax 96 microplate luminometer (Promega, USA). Promoter activity was normalized to Renilla luciferase activity.

### Quantitative real time PCR

Quantitative real-time PCR was performed using the StepOne Real-time PCR system (Applied Biosystems, USA). RNA was isolated from treated cells using Qiazol (Qiagen, Netherlands), according to the manufacturer's instructions. Synthesis of complementary DNA used 2 μg RNA and 2-4 μl of this was amplified using the KAPA SYBR Fast mastermix (KAPA Biosystems, South Africa) for qRT-PCR analysis. The sequence of qRT-PCR primers were as follows; MMP-2 F: 5′ TGGCGATGGATACCCCTTT 3′, R: 5′ TTCTCCCAAGGTCCATAGCTCAT 3′, TIMP-1 F: 5′ AGAGACACCAGAGAACCCA 3′, R: 5′ TGATGACGAGGTCGGAATTG 3′, TIMP-2 F: 5′ CATGATCCCGTGCTACATCTC 3′, R: 5′ TTGATGC AGGCGAAGAACT 3′, IL-1β F: 5′ CCACCTCCAGG GACAGGATA 3′ and R: 5′ TGGGATCTACACTCTC CAGC 3′, IL-6 F: 5′ GGATTCAATGAGGAGACTTGCC 3′ and R: 5′ CAGGCTGGCATTTGTGGTTG 3′, TNF-α F: 5′ GTAGCCCATGTTGTAGCAAACC 3′ and R: 5′ TGA TGGCAGAGAGGAGGTTG 3′, GM-CSF F: 5′ GAC ACTGCTGCTGAGATGAATG 3′ and R: CAGTGC TGCTTGTAGTGGCT 3′, GAPDH F: 5′GGCTCT CCAGAACATCATCC 3′ and R: 5′ GCCTGCTTC ACCACCTTC 3′. Samples were standardized to the house-keeping gene, GAPDH. Analysis was carried out using the comparative threshold cycle (C_T_) method.

### Statistical analysis

Experiments were performed in triplicate and represented as mean ± SD and repeated at least two independent times unless stated otherwise. The student's *t* test was performed in GraphPad Prism V5.0 for all comparisons. A *p* value of p<0.05 was considered statistically significant.

## Abbreviations

KPNB1: Karyopherin beta 1
NLS: Nuclear localisation sequence
NPC: Nuclear pore complex
NFkB: Nuclear factor kappa B
AP-1: Activator protein 1
IL-1β: Interleukin 1 beta
IL-6: Interleukin 6
TNF-α: Tumour necrosis factor alpha
MMP: matrix metalloprotease
TIMP: Tissue inhibitor of metalloproteinase
GM-CSF: Granulocyte macrophage colony stimulating factor
JNK: cJUN N-terminal kinase
